# Hands-On Defibrillation Has the Potential to Improve the Quality of Cardiopulmonary Resuscitation and Is Safe for Rescuers—A Preclinical Study

**DOI:** 10.1161/JAHA.112.001313

**Published:** 2012-10-25

**Authors:** Tobias Neumann, Matthias Gruenewald, Christoph Lauenstein, Tobias Drews, Timo Iden, Patrick Meybohm

**Affiliations:** Department of Anaesthesiology and Intensive Care Medicine, University Hospital Schleswig-Holstein, Kiel, Germany (T.N., M.G., C.L., T.D., T.I., P.M.); Department of Anaesthesiology and Intensive Care Medicine, University Hospital of Cologne, Koeln, Germany (T.N.); Clinic of Anaesthesiology, Intensive Care Medicine and Pain Therapy, University Hospital Frankfurt am Main, Frankfurt am Main, Germany (P.M.)

**Keywords:** cardiac arrest, cardiopulmonary resuscitation, chest compression, defibrillation, resuscitation

## Abstract

**Background:**

Recently, it has been demonstrated that rescuers could safely provide a low, static downward force in direct contact with patients during elective cardioversion. The purpose of our experimental study was to investigate whether shock delivery during uninterrupted chest compressions may have an impact on cardiopulmonary resuscitation (CPR) quality and can be safely performed in a realistic animal model of CPR.

**Methods and Results:**

Twenty anesthetized swine were subjected to 7 minutes of ventricular fibrillation followed by CPR according to the 2010 American Heart Association Guidelines. Pregelled self-adhesive defibrillation electrodes were attached onto the torso in the ventrodorsal direction and connected to a biphasic defibrillator. Animals were randomized either to (1) hands-on defibrillation, where rescuers wore 2 pairs of polyethylene gloves and shocks were delivered during ongoing chest compressions, or (2) hands-off defibrillation, where hands were taken off during defibrillation. CPR was successful in 9 out of 10 animals in the hands-on group (versus 8 out of 10 animals in the hands-off group; not significant). In the hands-on group, chest compressions were interrupted for 0.8% [0.6%; 1.4%] of the total CPR time (versus 8.2% [4.2%; 9.0%]; *P*=0.0003), and coronary perfusion pressure was earlier restored to its pre-interruption level (*P*=0.0205). Also, rescuers neither sensed any kind of electric stimulus nor did Holter ECG reveal any serious cardiac arrhythmia.

**Conclusions:**

Hands-on defibrillation may improve CPR quality and could be safely performed during uninterrupted chest compressions in our standardized porcine model.

## Introduction

For many decades, it has been a well-trained practice to make sure that hands are “off” during standard defibrillation and shock delivery. But in clearing the patient, chest compressions are necessarily interrupted.^1^ On the other hand, the quality of cardiopulmonary resuscitation (CPR) is crucial, and therefore, the latest resuscitation guidelines emphasized minimization of chest compression interruptions^2,3^ and identified reduction of no flow time as an important issue of CPR quality.^4^ One key to more success in CPR quality may be uninterrupted chest compressions with fewer “Hands-Off” phases in the context of shock delivery.

Defibrillation technology has been improved over recent years, as external defibrillators provide a biphasic waveform impulse within milliseconds with reduced peak voltages. In addition, pregelled self-adhesive electrode pads provide adequate electrical contact to the patient and more safety for the rescuer compared with handheld paddles because of their nonconductive backing.

Recently, Lloyd et al demonstrated that elective cardioversion could be safely performed in men when rescuers were in direct manual contact with the patient, providing a low, static downward force of 9.1 kg (20 pounds).^5^ Furthermore, the average leakage current flowing through the rescuer's body for each phase of the defibrillation impulse was below several recommended safety standards for leakage current, and none of the shocks was perceptible to the rescuer. However, this study was limited by the artificial circumstances and does not represent a realistic scenario of CPR. A systematic literature search previously revealed a total of 29 adverse events that were reported with tingling sensations and minor burns as consequences of inadvertent shocks; however, no case report could be identified in which medical personnel or bystanders sustained a life-threatening condition.^6^

The purpose of our experimental study was to investigate whether hands-on defibrillation combined with uninterrupted chest compressions may have an impact on CPR quality and can be safely performed in a realistic scenario of CPR.

## Methods

This was an experimental study on 20 healthy Goettingen miniature pigs aged 1 to 3 years of both sexes weighing 34.5 kg (30.8 kg; 38.9 kg). The project was approved by the Animal Investigation Committee of the Christian-Albrechts University of Kiel, Germany, and animals were managed in accordance with institutional guidelines and the Utstein-style guidelines.^7^ All animals received human care in compliance with the “Guide for the Care and Use of Laboratory Animals,” published by the National Institutes of Health (NIH Publication No. 88.23, revised 1996). For reporting our results, we considered the ARRIVE (Animals in Research: Reporting In Vivo Experiments) guidelines.^8^

All investigators performing chest compressions in this study were healthcare professionals with knowledge and experience of the risks of defibrillation attempts. They all were informed about currently available case reports on defibrillator misuse or malfunction^6^ and about the findings of Lloyd and his team.^5^ Informed consent of the investigators was implicit in the design of the study.

### Animal Preparation

The animals were fasted overnight but had free access to water. Anesthesia was initiated by intramuscular injection of azaperone (2 mg/kg), esketamine (1 mg/kg), and atropine (0.02 mg/kg) and was completed by ear vein injection of propofol (1 to 2 mg/kg) and sufentanil (0.3 μg/kg). After endotracheal intubation, pigs were ventilated with a volume-controlled ventilator (tidal volume 10 mL/kg, FiO_2_ 0.3, positive end-expiratory pressure 5 mmHg). To maintain normocapnia, the respiratory rate (15 to 20 per minute) was adjusted to end-tidal carbon dioxide (etCO_2_) that was monitored by an inspired/expired gas analyzer. Intravenous anesthesia was maintained by continuous infusion of propofol (4 to 6 mg/kg per hour) and sufentanil (0.5 μg/kg per hour). Ringer's solution (10 mL/kg per hour) was administered continuously throughout the preparation phase to replace fluid loss during instrumentation. A standard leads II and V_5_ electrocardiogram was used to monitor heart rhythm. Depth of anesthesia was judged according to blood pressure and heart rate. Swine do not respond to painful or auditory stimuli under this anesthetic regimen.

A 7 F saline-filled central venous catheter was inserted in the left internal jugular vein for drug administration and a 7 F saline-filled sheath contralaterally. A 4 F catheter was inserted percutaneously into the right femoral artery to determine mean arterial blood pressure. Intravascular catheters were attached to pressure transducers that were aligned at the level of the right atrium. All catheters were flushed with isotonic saline containing 10 IU/mL heparin at a rate of 3 mL per hour to prevent obstruction. Normothermic body temperature was maintained at 311 K (100.4 °F or 37.9 °C) in all animals with a heating blanket throughout the study period.

### Defibrillation Protocol

**Figure 1.**
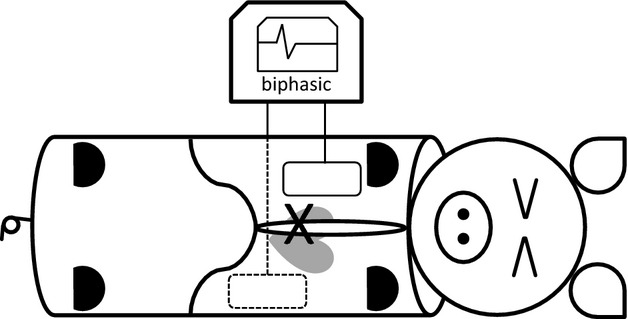
Experimental setup. One defibrillation electrode was attached on the right half of the anterior thorax and a second electrode in a posterolateral position on the left thorax. Both electrodes were connected to a biphasic defibrillator. The cross indicates placement of rescuer's hands.

Pregelled self-adhesive defibrillation electrodes with nonconductive backing (corPatch easy, Leonhard Lang GmbH, Innsbruck, Austria) were attached onto the torso in the ventrodorsal direction (1 electrode on the right half of the anterior thorax and a second electrode in a posterolateral position on the left thorax) and connected to a biphasic defibrillator (corpuls3, GS Elektromed. Geräte G. Stemple GmbH, Kaufering, Germany; Figure 1). Transthoracic impedance was measured by the defibrillator. Animals were randomized by using closed envelopes before the beginning of the project either to (1) hands-on defibrillation, where rescuers wore 2 pairs of usual medical polyethylene gloves and defibrillation was delivered during ongoing chest compressions or (2) hands-off defibrillation, where hands were taken off during defibrillation. In the hands-on group, chest compressions were interrupted for brief rhythm analysis only when either a marked increase of arterial blood pressure or etCO_2_ was observed, suggesting spontaneous circulation.

We provide a video file in the Online Data Supplement, which provides an example of hands-on defibrillation and successful resuscitation.

Two investigators were prepared as rescuers and randomly assigned to begin resuscitation by manual chest compressions. Switching the compressor was performed every 4 minutes lasting less than 2 seconds. Rescuers wore usual operating-theater clogs (designated as antistatic according to EN standard 344) standing on a metallic surgical step stool with the upper part of their bodies positioned above the pig's torso. To perform chest compressions safely — also during hands-on defibrillation — rescuers wore 2 pairs of usual polyethylene examination gloves, as undetected glove lesions could not fully be excluded. Except for the rescuer's gloved hands, there was no other electrical contact with the pig, especially no wet conditions. The designated rescuers were connected to a Holter ECG (CardioDay Holter ECG, Getemed, Teltow, Germany) in order to record heart rate and to analyze potential cardiac arrhythmia. Standard leads I and II were obtained. In addition, all recordings were reviewed and edited by a well-trained cardiologist blinded to the treatment group. The total number of potential premature atrial contractions, premature ventricular contractions, bigeminy, and salvos was counted.

### Experimental Protocol

**Figure 2.**
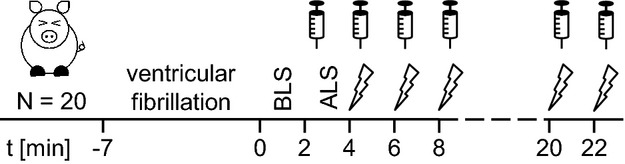
Experimental timeline. Animals were prepared and fibrillated under general anesthesia. After 7 minutes of cardiac arrest, all animals received CPR starting with 2 minutes of basic life support (BLS) and subsequent advanced life support (ALS) with alternating administration of epinephrine and vasopressin. After 4 minutes of CPR, all animals were defibrillated every 2 minutes.

Following hemodynamic measurements at baseline, ventricular fibrillation was electrically induced by an alternating current of 5 to 10 V and 1 to 2 mA by a 5 F pacing catheter. Mechanical ventilation and anesthesia were discontinued after cardiac arrest, which was identified by a ventricular fibrillation pattern on the ECG and a systolic arterial blood pressure <25 mmHg. To prevent clot formation, the animals received heparin (100 IU/kg) prior to induction of cardiac arrest. After a 7-minute nonintervention interval of untreated ventricular fibrillation, basic life support CPR was simulated for 2 minutes, applying external manual chest compressions at a rate of 100 per minute with a 50% duty cycle, a compression depth of 25% of the anterior–posterior diameter of the chest wall, and ventilations with 100% oxygen at 12 breaths per minute. Subsequently, advanced cardiac life support was started with alternating administration of 15 μg/kg epinephrine and 0.3 IU/kg vasopressin every second minute. The first biphasic defibrillation attempt was administered after a total of 4 minutes of chest compressions at 3 J/kg according to the 1-shock protocol and was repeated every 2 minutes with increasing energy up to 4 J/kg as suggested by the AHA guidelines^9^ (Figure 2). Return of spontaneous circulation (ROSC) was defined as maintenance of an unassisted pulse and a systolic aortic blood pressure ≥60 mm Hg lasting for 10 consecutive minutes according to the Utstein-style guidelines.^7^ CPR was terminated when resuscitation remained unsuccessful for 23 minutes.

After ROSC anesthesia was restarted by continuous infusion of propofol (4 to 6 mg/kg per hour) and sufentanil (0.5 μg/kg per hour). The FiO_2_ was reduced 15 minutes after ROSC to 0.5 to avoid hyperoxia and further decreased to maintain the saturation of peripheral oxygenation (SpO_2_) between 94% and 96%. During the initial postresuscitation period, animals received crystalloid infusions to keep mean arterial blood pressure above 50 mm Hg and central venous pressure above 5 mmHg. If this first step failed, additional (nor)-epinephrine was administered to keep mean arterial blood pressure above 50 mmHg. Four hours after ROSC, animals were euthanized by an overdose of sufentanil, propofol, and potassium chloride. Postmortem examination was routinely performed for documentation of potential injuries to the thoracic and abdominal cavities during CPR.

### Measurements

#### CPR comparability measures

To confirm that both groups received similar CPR except for the defibrillation technique, the CPR time to ROSC, number of shocks, cumulative defibrillation energy, and vasopressor doses as well as end-tidal carbon dioxide, blood lactate, and pH (measured by an automatic blood gas analyzer: GEM 4000, Instrumentation Laboratory GmbH, Munich, Germany) were recorded during CPR.

#### CPR efficacy measures

**Figure 3.**
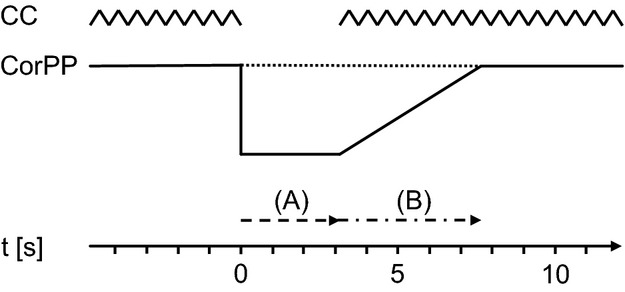
Illustration of CPR efficacy measures. After every interruption of chest compressions (CCs), several compressions are needed to restore coronary perfusion pressure (CorPP) back to its preinterruption level. For our efficacy end points, we measured both (A) the interval of the interruption (no flow time and (B) the duration of the restoration of CorPP (restoration time).

We defined the number of ROSC per group, the no flow ratio, and the coronary perfusion pressure (CorPP) restoration ratio as efficacy measures. The no flow ratio was calculated as the ratio of cumulative chest compression interruption intervals divided by the total CPR time.^10^ Since it needs several chest compressions to restore CorPP to its pre-interruption level, restoration time was calculated as the interval from restarting CPR to the moment when CorPP reaches its pre-interruption level (Fig. 3). If restoration failed, the restoration time was defined as the interval from restarting CPR to the next interruption. CorPP restoration ratio was calculated as the cumulative CorPP restoration time divided by the total CPR time. Further, it was tested whether lactate distribution (washout) differed between groups.

#### CPR safety measures

As safety measures, we defined for the rescuer the perception of any inadvertent event due to defibrillation as well as cardiac arrhythmia (detected in Holter ECG recordings within 12 hours after resuscitation). As an important parameter of current conduction, transthoracic impedance in swine was measured by the defibrillator. Postmortem examination of the thoracic and abdominal cavities of the swine was carried out for detection of any injury.

### Statistical Analysis

Data from each resuscitation episode were collected and are expressed as median [25%; 75% quartile] or scatter plot diagrams with medians unless otherwise specified. Statistics were performed using commercially available statistics software (GraphPad Prism version 5.01 for Windows, GraphPad Software Inc, San Diego, CA). Survival rates were compared using Fisher's exact test taking dichotomous variables (treatment groups and survival) and 2-sided *P* values. Data for CPR comparability as well as no flow and restoration times were analyzed by the Mann–Whitney *U* test for nonparametric and unpaired data. CPR time to ROSC was compared by survival analysis with a subsequent log-rank test. Distribution of blood lactate over time was compared by ANOVA (2-way). Results were considered statistically significant at *P*≤0.05. The authors had full access to the data and take full responsibility for its integrity.

## Results

### CPR Comparability Measures

**Figure 4.**
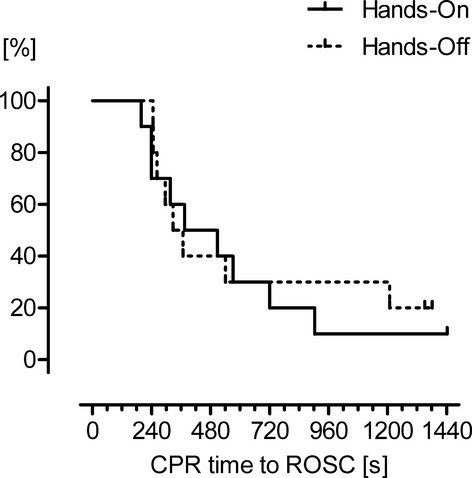
Each drop in these survival curves indicates return of spontaneous circulation (ROSC). Survival analysis did not reveal significant differences between groups in the duration of cardiopulmonary resuscitation (log-rank test, *P*=0.7021). In prolonged resuscitation swine tend to benefit from hands-on technique.

CPR data in groups were comparable (eg, number of shocks, cumulative vasopressor dose, and etCO_2_) and did not differ significantly. Survival analysis for time to ROSC did not reveal significant differences between groups (log-rank test, *P*=0.7021). Survival curves illustrate CPR time to ROSC in both groups (Figure 4). Detailed data of treatment groups are presented in Table 1.

**Table 1.** Comparability of Cardiopulmonary Resuscitation Data

	Hands-On (n=7)	Hands-Off (n=8)	*P*
CPR time to ROSC, s	374 [239; 646]	312 [251; 498]	0.9616

Number of shocks, n	4 [3; 9]	3.5 [2.25; 9.75]	0.9085

Cumulative defibrillation energy, J	640 [440; 1640]	540 [290; 1790]	0.8667

Cumulative epinephrine dose, μg/kg	33 [27; 68]	42 [28; 134]	0.5338

Cumulative vasopressin dose, IU/kg	0.70 [0.48; 0.82]	0.51 [0.31; 0.64]	0.1893

etCO_2_ 5 min CPR, mmHg	19 [8; 24]	13 [9; 19.5]	0.6249

Blood lactate 5 min CPR, mmol/L	8.7 [4.4; 10.1]	5.0 [4.5; 5.3]	0.1331

pH 5 min CPR	7.29 [7.16; 7.59]	7.47 [7.35; 7.58]	0.4452

Cardiopulmonary resuscitation (CPR) time to return of spontaneous circulation (ROSC), number of shocks, cumulative defibrillation energy, and cumulative vasopressor dose did not differ significantly between groups, as well as end-tidal carbon dioxide (etCO_2_), lactate, and pH, that were assessed 5 minutes after start of CPR. Data are median (25%; 75% quartiles). Three animals in the hands-on and 2 animals in the hands-off group that received only 1 shock were excluded from analysis.

### CPR Efficacy Measures

ROSC was achieved in 9 of 10 animals in the hands-on group compared with 8 of 10 animals in the hands-off group (not significant).

**Figure 5.**
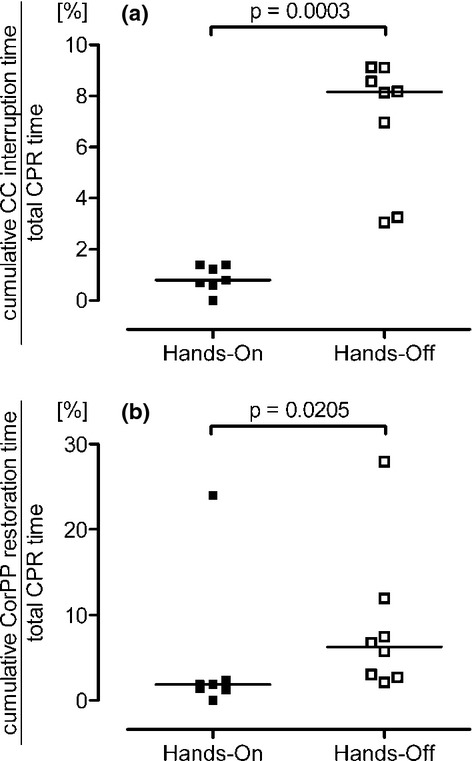
Results of CPR efficacy measures. No flow time as the sum of all chest compression (CC) interruption intervals related to total cardiopulmonary resuscitation (CPR) time (A) and restoration ratio of coronary perfusion pressure (CorPP) as the ratio of cumulative restoration time of CorPP to total CPR time (B) for each swine that received >1 shock (hands-on group n=7, hands-off group n=8; we excluded from further analysis 3 animals in the hands-on group and 2 animals in the hands-off group that received only 1 shock). Scatter plots with medians are shown.

**Figure 6.**
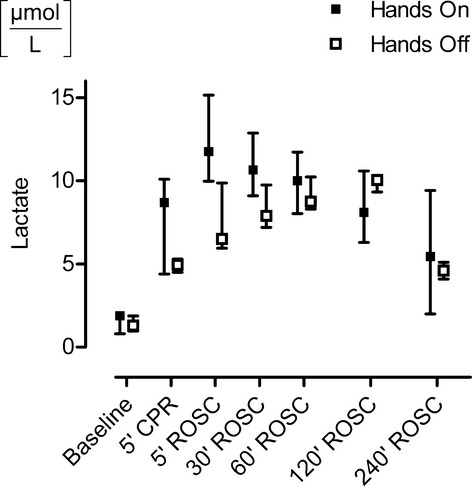
Blood lactate distribution over time. To show the efficacy of removal of lactate sequestered in tissue into central circulation, we determined lactate level at baseline, 5 minutes after initiation of cardiopulmonary resuscitation (CPR), and 5, 30, 60, 120, and 240 minutes after return of spontaneous circulation (ROSC). Peak of lactate appeared much earlier in the hands-on group, suggesting a more intensive reperfusion shortly after ROSC. Error bars show interquartile range.

To analyze differences between hands-on and hands-off defibrillation, it was necessary to exclude animals that responded to the first defibrillation attempt with ROSC from further analysis (3 animals hands-on, 2 animals hands-off). For example, swine that receive just 1 shock will plausibly have a restoration time of 0 regardless of the used defibrillation method, as chest compressions were immediately stopped because of increasing blood pressure or etCO_2_. In the hands-on group, chest compressions were interrupted for 0.8% [0.6%; 1.4%] of the total CPR time (versus 8.2% [4.2%; 9.0%] in the hands-off group; *P*=0.0003). The restoration ratio of CorPP was significantly lower in the hands-on group (1.9% [1.3%; 2.4%] of the total CPR time) compared with the hands-off group (6.3% [2.8%; 10.8%] of the total CPR time; *P*=0.0205). Individual no flow times and restoration times are plotted in Figure 5. Lactate distribution over time did not significantly differ between groups and is presented in Figure 6. However, peak lactate concentration appeared earlier in the hands-on group (5 minutes after ROSC) compared with the hands-off group (2 hours after ROSC).

In the hands-on group, interruptions for rhythm analysis were performed 34 times when either a marked increase in blood pressure or etCO_2_ was observed. Spontaneous circulation could be confirmed 13 times (38%).

### CPR Safety Measures

Overall, 37 shocks were delivered in the hands-on group. Rescuers felt the muscular contraction of the pig's body but never any kind of electric stimulus. Holter ECG analysis did not reveal any serious cardiac arrhythmia event in any rescuer (Table 2). Transthoracic impedance did not differ significantly between both groups (64 [36; 86] Ω versus 64 [39; 80] Ω; *P*=0.7231).

**Table 2.** Safety Measure: Rescuer's Holter ECG Data

	Hands On	Hands Off	*P*
Premature atrial contractions (PACs) per hour	3.3 [0.2; 9.3] 5.0±5.4	3.3 [0.7; 5.3] 3.1±2.4	0.8253

Premature ventricular contractions (PVCs) per hour	0.0 [0.0; 0.0] 0.2±0.5	0.0 [0.0; 1.5] 0.6±1.3	0.7270

Heart rate, range	46 to 132	41 to 95	

The coinvestigators performing chest compressions were connected to a Holter ECG. The records did not reveal any serious cardiac arrhythmias. Neither bigeminy nor salvos were detected. Data are expressed as median [25%; 75% quartile] and mean±SD and for heart rate as the range from lowest to highest observed heart rate.

## Discussion

During CPR, “Hands-Off” periods are traditionally used to prevent potential harm to rescuers during defibrillation and for rhythm analysis, but these pauses interfere with current recommendations emphasizing the importance of chest compressions. Little attention has yet been paid to strategies to further reduce pauses, for example, by uninterrupted manual chest compressions during defibrillation.

We have shown—to the best of our knowledge for the first time—that hands-on defibrillation (1) shortens CPR pauses, (2) hastens restoration of preinterruption coronary perfusion pressure, and (3) could be safely performed in a realistic porcine model of CPR.

### CPR Quality

The adverse hemodynamic consequences of delays and pauses between chest compression and defibrillation have been clearly demonstrated.^11^ In our animal model, preventing hands-off periods shortened CPR pauses and hastened restoration of coronary perfusion pressure, thereby improving the quality of chest compressions. Moreover, the peak of lactate was observed much earlier in the hands-on group, suggesting a more intensive reperfusion 5 minutes after ROSC. However, we did not find any difference in terms of ROSC rate, as this study was not designed or powered to find differences between survivors and nonsurvivors but rather to describe CPR quality. To show more clearly the advantages of hands-on defibrillation, further animal studies should probably use a longer period of cardiac arrest, as survival curves show a tendency that hands-on defibrillation may be superior in prolonged resuscitation (Figure 4).

Although no flow time was extremely short in the hands-on group, no flow time was also very short in the hands-off group, probably because of the strict standardization in our experimental setting, as chest compressions were interrupted for only 8.2% of the total CPR time. In many clinical studies, however, reported “Hands-Off” no flow times are much longer, ranging between 12% and 48%.^12–14^ Thus, one may speculate that the difference in resuscitation quality and effectiveness between hands-on and hands-off techniques may even become more relevant in clinical practice. Furthermore, uninterrupted chest compressions would at least represent a protocol simplification of CPR.

From our experience, however, hands-on defibrillation raises an important issue: *How can successful resuscitation reliably be detected during uninterrupted chest compressions such that CPR can be stopped?* There have recently been substantial improvements in external defibrillation technology. Enhancements of ECG filtering may permit rhythm analysis during chest compressions, which unfortunately was not implemented in the defibrillator used in our study. Because chest compressions caused motion artifacts in the ECG, resulting in difficulties recognizing the correct rhythm, we defined potential resuscitation success by a marked increase of either blood pressure or etCO_2_ during CPR. Concerning these conditions, interruptions for rhythm analysis were performed 34 times in the hands-on group, but retrospectively 62% of all interruptions in the hands-on group were unnecessary. On the other hand, we also cannot exclude that some swine may have gained spontaneous circulation much earlier, for example, in the case of an undetected successful shock attempt during ongoing chest compressions. Therefore, more sophisticated ECG filter software integrated into defibrillators may be needed to enable analysis of ECG rhythms during ongoing chest compressions and to avoid unnecessary pauses. Berger and colleagues previously developed a motion artifact reduction system, based on adaptive noise cancellation techniques, that allowed automated rhythm discrimination during uninterrupted CPR.^15^ In addition, Li et al recently presented a wavelet-based transformation and shape-based morphology detection for ECG rhythm classification.^16^

### CPR Safety

Induction of cardiac arrhythmia and ventricular fibrillation in the rescuer himself or herself is certainly the most feared complication during defibrillation. However, leakage current varies widely depending on the type of equipment and exposure. Given a certain discharge voltage of the defibrillator, the lower the skin impedance of the patient (eg, by good electrode contact) and the higher the impedance of the rescuer (eg, by wearing polyethylene gloves), the lower the leakage current through the rescuer. A recent review by Petley et al explained the physical principles of hands-on defibrillation in a very detailed manner.^17^ In our study, median transthoracic impedance was 64Ω in both groups, reasonably comparable to human data and suggesting good electrical contact.^18–20^ We did not measure intracardiac current flow in swine. Because transthoracic impedance and the used defibrillation energies did not significantly differ between the groups, we hypothesized that, according to Ohm's law, intracardiac current might not be significantly different either. Modern external defibrillators with biphasic shocks and real-time impedance compensation technologies adjusting either voltage or phase duration have previously reduced peak voltages and impulse duration. Furthermore, paddles have been replaced in many settings by defibrillation electrodes, which result in better and more consistent electrode–skin coupling. These circumstances provide the basis for a safe application of hands-on defibrillation during continuous manual chest compressions throughout the resuscitation period. Within the constraints of our model, none of the 37 shocks was perceptible as an electric stimulus. This is supported by the findings of Lloyd et al that none of the volunteers sensed the shocks because the maximum value of leakage current was below a well-accepted threshold of perception.^5^ In addition, Holter ECG analysis did not reveal any serious cardiac arrhythmia event in any rescuer. The registered premature contractions were within a physiological range.

There are several points that may have limited our results: First, both long-term survival and neurological outcome were not evaluated because of limitations posed by governmental regulations. Second, blinding the investigators was not possible throughout the experiment, but hemodynamic variables, blood gases, and Holter ECG data were analyzed in a blinded fashion. Third, we used self-adhesive defibrillation electrodes and rescuers wore examination gloves. Before defibrillation protocols can be changed, however, more definitive data are needed to make absolutely sure there is no risk. Such studies have to focus on different scenarios including handheld paddles, miscellaneous defibrillator technologies, high-impedance patients, wet and metal surfaces, no-gloves situations, and resuscitation scenarios within limited space. Because we observed a pronounced effect on reduction of no flow time, we also encourage investigation into other technical solutions for safe application of hands-on defibrillation strategy. We emphasize that the safety of rescuers is an absolute requisite. Therefore, we recommend the safety checklist from our laboratory (Table 3). Fourth, defibrillation caused an intense contraction of the pig's skeletal muscles. Although we did not observe severe injuries in either swine or rescuers, it is imaginable that in humans there might be a potential risk of trauma to the patient or the rescuer in the case of a simultaneous forcible compression and an intense contraction of the patient's body. Fifth, hands-on defibrillation is currently off-label use. Therefore, further studies are urgently needed before hands-on defibrillation can be incorporated into daily routine.

**Table 3.** Safety Checklist

Perform hands-on defibrillation only if you can check off every item!

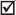 Wearing polyethylene gloves?

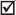 Biphasic defibrillator?

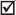 Self-adhesive defibrillation electrodes with nonconductive backing?

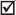 Electrodes in good contact with patient's skin?

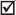 No conductive circumstances (eg, body fluids, intravenous fluids)?

For further studies on hands-on defibrillation, we recommend the safety checklist used in our laboratory.

Because most of the existing case reports about accidental shocks to responders or bystanders do not give detailed information about the circumstances, we will soon provide an online register at http://www.hands-on-cpr.net for the optional use of recording adverse events of defibrillation attempts in a structured manner.

## Conclusions

Uninterrupted manual chest compressions during shock delivery are feasible. Considering the limitations of our experimental model, hands-on defibrillation shortens CPR pauses and hastens restoration of coronary perfusion pressure, which is known to be predictive of resuscitation outcome. Hands-on defibrillation may have the potential to eliminate hazardous delays from “all-clear” periods during resuscitation and may thereby improve CPR quality. However, inadequate ECG rhythm analysis during CPR is still a limiting factor. To further reduce unnecessary interruptions, more sophisticated ECG filter software integrated into defibrillators is probably needed to enable adequate ECG analysis during ongoing chest compressions. We have demonstrated that hands-on defibrillation can be safely performed during ongoing chest compressions. Nevertheless, using a checklist of safety precautions should be recommended during hands-on defibrillation, and further studies are urgently needed before hands-on defibrillation can be implemented in clinical practice.

## References

[1] Yu T Weil MH Tang W Sun S Klouche K Povoas H Bisera J Adverse outcomes of interrupted precordial compression during automated defibrillation. Circulation. 2002;106:368-372. 12119255 10.1161/01.cir.0000021429.22005.2e

[2] Berg RA Hemphill R Abella BS Aufderheide TP Cave DM Hazinski MF Lerner EB Rea TD Sayre MR Swor RA Part 5: Adult basic life support: 2010 American Heart Association Guidelines for cardiopulmonary resuscitation and emergency cardiovascular care. Circulation. 2010;122:S685-S705. 20956221 10.1161/CIRCULATIONAHA.110.970939

[3] Sayre MR Berg RA Cave DM Page RL Potts J White RD Hands-only (compression-only) cardiopulmonary resuscitation: a call to action for bystander response to adults who experience out-of-hospital sudden cardiac arrest: a science advisory for the public from the American Heart Association Emergency Cardiovascular Care Committee. Circulation. 2008;117:2162-2167. 18378619 10.1161/CIRCULATIONAHA.107.189380

[4] Hazinski MF Nolan JP Billi JE Bottiger BW Bossaert L de Caen AR Deakin CD Drajer S Eigel B Hickey RW Jacobs I Kleinman ME Kloeck W Koster RW Lim SH Mancini ME Montgomery WH Morley PT Morrison LJ Nadkarni VM O'Connor RE Okada K Perlman JM Sayre MR Shuster M Soar J Sunde K Travers AH Wyllie J Zideman D Part 1: Executive summary: 2010 International Consensus on cardiopulmonary resuscitation and emergency cardiovascular care science with treatment recommendations. Circulation. 2010;122:S250-S275. 20956249 10.1161/CIRCULATIONAHA.110.970897

[5] Lloyd MS Heeke B Walter PF Langberg JJ Hands-on defibrillation: an analysis of electrical current flow through rescuers in direct contact with patients during biphasic external defibrillation. Circulation. 2008;117:2510-2514. 18458166 10.1161/CIRCULATIONAHA.107.763011

[6] Hoke RS Heinroth K Trappe HJ Werdan K Is external defibrillation an electric threat for bystanders?. Resuscitation. 2009;80:395-401. 19211180 10.1016/j.resuscitation.2009.01.002

[7] Idris AH Becker LB Ornato JP Hedges JR Bircher NG Chandra NC Cummins RO Dick W Ebmeyer U Halperin HR Hazinski MF Kerber RE Kern KB Safar P Steen PA Swindle MM Tsitlik JE von Planta I von Planta M Wears RL Weil MH Utstein-style guidelines for uniform reporting of laboratory CPR research. A statement for healthcare professionals from a Task Force of the American Heart Association, the American College of Emergency Physicians, the American College of Cardiology, the European Resuscitation Council, the Heart and Stroke Foundation of Canada, the Institute of Critical Care Medicine, the Safar Center for Resuscitation Research, and the Society for Academic Emergency Medicine. Circulation. 1996;94:2324-2336. 8901707 10.1161/01.cir.94.9.2324

[8] Kilkenny C Browne WJ Cuthill IC Emerson M Altman DG Improving bioscience research reporting: the ARRIVE guidelines for reporting animal research. PLoS Biol. 2000;8:e1000412 10.1371/journal.pbio.1000412 PMC2893951 20613859

[9] Neumar RW Otto CW Link MS Kronick SL Shuster M Callaway CW Kudenchuk PJ Ornato JP McNally B Silvers SM Passman RS White RD Hess EP Tang W Davis D Sinz E Morrison LJ Part 8: Adult advanced cardiovascular life support: 2010 American Heart Association Guidelines for cardiopulmonary resuscitation and emergency cardiovascular care. Circulation. 2010;122:S729-S767. 20956224 10.1161/CIRCULATIONAHA.110.970988

[10] Kramer-Johansen J Edelson DP Losert H Koehler K Abella BS Uniform reporting of measured quality of cardiopulmonary resuscitation (CPR). Resuscitation. 2007;74:406-417. 17391831 10.1016/j.resuscitation.2007.01.024

[11] Steen S Liao Q Pierre L Paskevicius A Sjoberg T The critical importance of minimal delay between chest compressions and subsequent defibrillation: a haemodynamic explanation. Resuscitation. 2003;58:249-258. 12969599 10.1016/s0300-9572(03)00265-x

[12] Losert H Sterz F Koehler K Sodeck G Fleischhackl R Eisenburger P Kliegel A Herkner H Myklebust H Nysaether J Laggner AN Quality of cardiopulmonary resuscitation among highly trained staff in an emergency department setting. Arch Intern Med. 2006;166:2375-2380. 17130392 10.1001/archinte.166.21.2375

[13] Abella BS Alvarado JP Myklebust H Edelson DP Barry A O'Hearn N Vanden Hoek TL Becker LB Quality of cardiopulmonary resuscitation during in-hospital cardiac arrest. JAMA. 2005;293:305-310. 15657323 10.1001/jama.293.3.305

[14] Wik L Kramer-Johansen J Myklebust H Sorebo H Svensson L Fellows B Steen PA Quality of cardiopulmonary resuscitation during out-of-hospital cardiac arrest. JAMA. 2005;293:299-304. 15657322 10.1001/jama.293.3.299

[15] Berger RD Palazzolo J Halperin H Rhythm discrimination during uninterrupted CPR using motion artifact reduction system. Resuscitation. 2007;75:145-152. 17467872 10.1016/j.resuscitation.2007.03.007

[16] Li Y Bisera J Geheb F Tang W Weil MH Identifying potentially shockable rhythms without interrupting cardiopulmonary resuscitation. Crit Care Med. 2008;36:198-203. 18090359 10.1097/01.CCM.0000295589.64729.6B

[17] Petley GW Cottona AM Deakin CD Hands-on defibrillation: theoretical and practical aspects of patient and rescuer safety. Resuscitation. 2012;83:551-556. 22094984 10.1016/j.resuscitation.2011.11.005

[18] Kerber RE Martins JB Kienzle MG Constantin L Olshansky B Hopson R Charbonnier F Energy, current, and success in defibrillation and cardioversion: clinical studies using an automated impedance-based method of energy adjustment. Circulation. 1988;77:1038-1046. 3359585 10.1161/01.cir.77.5.1038

[19] Poole JE White RD Kanz KG Hengstenberg F Jarrard GT Robinson JC Santana V McKenas DK Rich N Rosas S Merritt S Magnotto L Gallagher JV III Gliner BE Jorgenson DB Morgan CB Dillon SM Kronmal RA Bardy GH for the LIFE Investigators Low-energy impedance-compensating biphasic waveforms terminate ventricular fibrillation at high rates in victims of out-of-hospital cardiac arrest. J Cardiovasc Electrophysiol. 1997;8:1373-1385. 9436775 10.1111/j.1540-8167.1997.tb01034.x

[20] Connell PN Ewy GA Dahl CF Ewy MD Transthoracic impedance to defibrillator discharge. Effect of electrode size and electrode-chest wall interface. J Electrocardiol. 1973;6:313-317. 4765325 10.1016/s0022-0736(73)80053-6

